# The Interplay Between lncRNAs–microRNAs Network Dysregulation and Cellular Hallmarks of Thyroid Cancer

**DOI:** 10.3390/cancers17203373

**Published:** 2025-10-18

**Authors:** Maryam Hejazi, Ramin Heshmat, Gita Shafiee, Bagher Larijani, Amir Ali Mokhtarzadeh, Vida Ebrahimi, Seyed Mohammad Tavangar

**Affiliations:** 1 Chronic Diseases Research Center, Endocrinology and Metabolism Population Sciences Institute, Tehran University of Medical Sciences, Tehran 1411713137, Iran; hejazi-m@tums.ac.ir (M.H.); rheshmat@tums.ac.ir (R.H.); g-shafiee@tums.ac.ir (G.S.); 2 Immunology Research Center, Tabriz University of Medical Sciences, Tabriz 5166614731, Iran; mokhtarzadehah@tbzmed.ac.ir; 3 Endocrinology and Metabolism Research Center, Endocrinology and Metabolism Clinical Sciences Institute, Tehran University of Medical Sciences, Tehran 1411713137, Iran; larijanib@tums.ac.ir; 4 Department of Pathology, Dr. Shariati Hospital, Tehran University of Medical Sciences, Tehran 1411713135, Iran

**Keywords:** thyroid cancer, long non-coding RNAs (lncRNAs), hallmarks of cancer, molecular biomarkers, gene regulation

## Abstract

**Background/Objectives**: Thyroid cancer (TC) is the most common type of endocrine neoplasm and is increasing in incidence, particularly papillary thyroid carcinoma (PTC). Early-stage disease has a favorable prognosis; however, advanced forms, such as anaplastic thyroid carcinoma, complicate treatment. Long non-coding RNAs (lncRNAs), longer than 200 nucleotides and non-coding, together with microRNAs, have emerged as major regulators of TC pathogenesis. This review summarizes data on how dysregulated lncRNAs influence the hallmarks of cancer in thyroid malignancies. **Methods**: We reviewed the literature on the role of lncRNAs and microRNAs in TC, focusing on their functions as competing endogenous RNAs (ceRNAs), regulators of PI3K/AKT and Wnt/β-catenin pathways, and controllers of epigenetic alterations. **Results**: Dysregulated lncRNAs contribute to hallmarks including sustained growth, evading suppressors, resisting death, replicative immortality, angiogenesis, invasion, metabolic reprogramming, immune evasion, genomic instability, and tumor-promoting inflammation. ceRNA mechanisms amplify immune evasion by regulating checkpoint proteins and cytokines, altering immune cell activity. Altered lncRNA profiles correlate with aggressiveness, metastasis, and prognosis. Notable lncRNAs, such as H19, MALAT1, and DOCK9-AS2, dysregulate oncogenic pathways and represent potential biomarkers. **Conclusions**: Advances in therapeutics suggest inhibiting oncogenic lncRNAs or restoring tumor-suppressive lncRNAs via RNA interference, antisense oligonucleotides, or CRISPR/Cas9 editing. New technologies, including single-cell RNA sequencing and spatial transcriptomics, will improve understanding of heterogeneous lncRNA–microRNA networks in TC and support precision medicine. LncRNAs signify both molecular drivers and clinical targets for thyroid cancer.

## Simple Summary

Thyroid cancer is the most common type of endocrine tumor, and its aggressive forms remain difficult to treat. In recent years, researchers have discovered that two types of non-coding RNAs, called long non-coding RNAs (lncRNAs) and microRNAs, play an important role in the behavior of thyroid cancer cells. These molecules do not make proteins but instead regulate how other genes are switched on or off. When their normal balance is disturbed, they can drive cancer cells to grow faster, spread to other tissues, or escape the body’s immune defenses. This review explains how changes in lncRNA–microRNA networks are connected to the main features of thyroid cancer, such as uncontrolled growth, resistance to cell death, and altered metabolism. Understanding these networks may open the way to new diagnostic tools and personalized treatments that target RNA molecules, improving outcomes for patients with thyroid cancer.

## 1. Introduction

Thyroid cancer is the most prevalent type of endocrine cancer diagnosed globally, with approximately 821,214 individuals diagnosed worldwide in 2022, resulting in a crude rate of 10.4 per 100,000 and an age standardized incidence rate (ASIR) of 9.1 per 100,000. The number of deaths is relatively low by comparison, with approximately 47,507 deaths (with an age standardized mortality rate, ASMR, of 0.44 per 100,000). The burden of thyroid cancer is significantly higher among women (74.9% of all cases), with an ASIR of 13.6 per 100,000 versus 4.6 per 100,000 in men [1]. Over the last 20 years, the incidence of thyroid cancer has risen substantially in high and middle income areas, primarily due to an increase in the diagnosis of papillary thyroid carcinoma (PTC), with stable or decreased rates of follicular (FTC), medullary (MTC), and anaplastic thyroid carcinoma (ATC) [2]. Population-based studies from the U.S. have shown that thyroid cancer is now the thirteenth most common cancer in the U.S. (with almost 44,000 new cases over a year in 2022), and is more common in women; lifetime risk estimates for women in the U.S. are about 1 in 55, and for men, 1 in 149 [3]. In its 2022 classification system, the World Health Organization now distinguishes between differentiated (PTC and FTC), medullary, poorly differentiated, and anaplastic thyroid tumor types, while also including new entities, such as salivary type carcinomas of the thyroid and tumors with uncertain malignant potential, further enhancing diagnostic accuracy and prognostic stratification [4]. While five-year survival rates for localized and regional differentiated thyroid cancer are greater than 98% in high-income countries, survival of advanced disease and ATC declined dramatically, emphasizing that early detection and histotype-specific treatment is crucial [3].

Long non-coding RNAs (lncRNAs) are defined as RNA transcripts longer than >200 nucleotides that do not have protein-coding capacity, although the definition varies regarding the upper limit of 200–500 nt [5]. They are recognized as being generated by RNA polymerase II, which undergo canonical processing including 5′ capping, splicing and polyadenylation, but they usually have some distinct biogenetic variations compared to mRNAs, such as alternative splicing and a non-canonical 3′ end. LncRNAs are diverse in sequence and structure, and also display systemic tissue-specific and time-specific expressions; they can be produced from intergenic regions from within introns or as antisense transcripts overlapping protein coding genes [6].

The classification of lncRNAs typically involves a number of criteria, which include the following: genomic context (e.g., long intergenic lncRNAs, antisense, intronic), subcellular location (nuclear versus cytoplasmic), structural properties, and mode of action. Nuclear lncRNAs are typically involved in chromatin remodeling and epigenetic regulation, while cytoplasmic lncRNAs are involved in post-transcriptional regulation (including determination of mRNA stability, translation, or acting as competing endogenous RNAs (ceRNAs) that sponge miRNAs) [7].

At the functional level, lncRNAs participate in various cellular processes that include the following: transcriptional regulation (serving as scaffolds for chromatin modifiers to organize and regulate transcription, or facilitating RNA DNA triplexes) [8], post-transcriptional regulation (modulating the splicing, stability, and translation of mRNA) [7,9], epigenetic regulation (via recruit histone modifiers and assembling a 3D chromatin structure) [8], and higher-order organization of cells via nuclear biomolecular condensates [10]. he modular nature of lncRNA provides them with unique opportunities to act as molecular master regulators of gene expression patterns, cell cycle trajectory, genome stability and cellular differentiation pathways [7].

The mechanisms used by lncRNAs to complete their tasks are influenced by the modular properties of lncRNAs and their spatiotemporal dynamics, which can include the following: scaffolding multi-protein complexes with distinct activities or functions, providing a localized guided route for proteins to walk to, providing a decoy for transcription factors or miRNAs, and encasing a higher-order subnuclear structure. Overall, lncRNAs have gained considerable attention in research communities studying development and cellular homeostasis, and also disease processes like cancer and endocrine dysfunctions, therefore acting as promising biomarkers for disease and therapeutics targets [11].

LncRNAs have been shown to be a significant contributor to thyroid cancer pathogenesis, and recent studies have provided evidence that lncRNAs can also interact with microRNAs and are important mediators of anti-tumor immunity. LncRNAs can function as competing endogenous RNAs (ceRNAs), sequestering miRNAs that have overlapping microRNA response elements (MREs) from target genes [12]. This post-transcriptional co-regulation can extend to immune-related genes, such as lncRNAs that sponge tumor-suppressive miRNAs and upregulate immune checkpoint molecules and immunosuppressive cytokines, contributing to tumor immune escape [13]. Inflammation can also influence lncRNA gene expression to establish feedback loops that enhance immune evasion—as an example, the lncRNA TINCR is induced by interferon-gamma and epigenetically and post-transcriptionally silences miR-199a-5p to stabilize PD-L1 and dampen T cell activity [14]. Although studies directly related to thyroid cancer are still emerging, integrated analyses have shown that lncRNA–miRNA networks correlate with immune cell infiltration in thyroid tumors [12]. Recently, high expression of the lncRNA H19 in thyroid carcinoma was associated with increased levels of T cells, B cells, dendritic cells, neutrophils, and macrophage infiltration, as well as increased immune gene signatures [15]. These observations link the role of lncRNAs in thyroid cancer to the more general context of miRNA-mediated immune regulation in cancer, suggesting that lncRNA–miRNA crosstalk is an important regulator of tumor–immune interactions and an exciting target for therapy in thyroid cancer and other similar cancers.

The importance of understanding the dysregulation of long non-coding RNAs (lncRNAs) associated with thyroid cancer (TC) is paramount, due to their wide-ranging effects on tumor development. A number of lncRNAs have been consistently identified as highly expressed or under-expressed in papillary, follicular, and anaplastic thyroid carcinoma that participate in the activation of important oncogenic processes, including dysregulated cell proliferation, cell invasion, and epithelial–mesenchymal transition, through regulating central pathways of importance such as PI3K/AKT, and Wnt/β-catenin [16]. In addition to the mechanistic pathways, there is growing support for dysregulated lncRNAs as specific biomarkers of diagnosis and prognosis. Many lncRNA candidates are generated in tumor tissue, in serum, or urine, and are linked with tumor stage, lymph node metastases, as well as patient outcomes [17]. The potential of lncRNAs as biomarkers of disease status or prognosis is further supported by the prediction of recurrence and progression-free survival in cohorts of papillary thyroid carcinoma, based on lncRNA signatures linked to genomic instability [18]. Additionally, some lncRNAs (i.e., MIAT) were found to participate in TC as a competitive endogenous RNA (ceRNA), competing against tumor-suppressive miRNAs’ (e.g., miR-150 5p) ectopic elevation of oncogenic driver expression such as EZH2, adding another dimension to the molecular understanding of the TC-associated lncRNA-mediated regulatory networks [19]. In summary, the strong evidence for lncRNAs participating in signaling, providing epigenetic control, clinical stratification, and ceRNA-mediated repression creates a need for essential studies investigating lncRNA dysregulation in thyroid cancer to find new diagnostic and therapeutic opportunities.

Recent evidence shows that ncRNA regulatory circuitry, such as miRNAs and lncRNAs, integrate into major oncogenic signaling pathways such as Hippo and MAPK, regulating proliferation, stemness, and therapy resistance in various cancers, including thyroid cancers [20].

Here, we review how dysregulated lncRNAs affect the signaling processes of thyroid cancer, in view of the established hallmarks of cancer as identified by Hanahan and Weinberg [21]. By focusing on a potential role for specific lncRNAs in contributing to key oncogenic features, including sustained proliferative signaling, evasion of growth suppressors, resistance to cell death, replicative immortality, angiogenesis, invasion and metastasis, metabolic reprogramming, immune evasion, genomic instability, and tumor-promoting inflammation, we provide a logical structure to understand the multifaceted contributions of lncRNAs in thyroid tumorigenesis. In addition, we emphasize the need to clarify the role of lncRNAs as clinical diagnostic or prognostic biomarkers, and relatively new therapeutic targets in thyroid cancer, and to consider future directions for the timely implementation of lncRNA-based studies in thyroid cancer patient-individualized medicine.

## 2. Materials and Methods

This paper is a narrative review derived from a search of the PubMed, Scopus, and Web of Science databases, with the last search conducted until September 2025. The search generated relevant articles regarding the roles of long non-coding RNAs (lncRNA) and microRNAs (miRNA) in thyroid cancer. The relevant studies identified received an initial screening and subsequent critical evaluation. This review only included peer-reviewed articles in English. As this is a literature-based study, the authors produced no new datasets, biospecimens, or studies. Therefore, ethical approval and patient consent were not necessary. All aspects of the review are supported by published articles, all of which are provided in the reference list. The authors declare that there are no restrictions on the availability of materials or information. The authors employed generative artificial intelligence (GenAI) for language editing and format improvement purposes only, and not for data generation, analysis, or interpretation.

## 3. Results

### 3.1. LncRNAs and the Hallmarks of Cancer in Thyroid Cancer

The original hallmarks of cancer described by Hanahan and Weinberg provide a useful framework to examine the variety of ways lncRNAs may contribute to thyroid tumorigenesis. In TC, the lncRNAs are responsible for maintaining proliferative signaling by regulating important growth pathways. For instance, lncRNA H19 is overexpressed in PTC and facilitates cell proliferation by activation of the PI3K/AKT pathway, in which plated silencing of lncRNA H19 showed a decrease in cell growth and increased apoptosis [17]. Evasion of growth suppressors is a hallmark that enables lncRNAs to impair tumor-suppressive biological networks, such as the p53 pathway. The lncRNA ZFAS1 is negatively regulated by p53-associated microRNAs, where its overexpression in PTC impedes upon p53-mediated growth arrest [22]. In the ability to resist cell death, lncRNAs are able to function as inhibitors of apoptosis in thyroid tumor cells. Knocking down lncRNA H19 upregulated the pro-apoptotic proteins Bax and caspase-3, whilst attenuating the oligomerization of PI3K/AKT, thereby inducing cell death in thyroid cancer cell lines [23]. The capability for replicative immortality is mediated by lncRNAs via their regulation of telomerase reverse transcriptase (TERT) expression. In the context of TC, several lncRNAs increase TERT activity and retain telomere length, bestowing replicative potential to the cells [24]. LncRNAs also regulate the induction of angiogenesis, with MALAT1 in particular enhancing neovascularization by promoting FGF2 secretion from tumor-associated macrophages in thyroid cancer, which supported tumor growth and metastatic potential [25]. In the descriptor for invasion and metastasis, the lncRNAs regulate the epithelial–mesenchymal transition (EMT) and dissemination of tumor cells. The exosomal lncRNA DOCK9-AS2 enhances EMT and stemness in PTC by modulating the Wnt/β-catenin pathway while interacting with SP1/miR-1972 signaling to support an aggressive tumor phenotype [26]. With respect to the deregulation of cellular energetics, lncRNAs drive the metabolic reprogramming of thyroid cancer cells to accommodate rapid tumor growth. Recently, the lncRNA PVT1 was shown to support glycolytic flux and mitochondrial function in endocrine tumors at least in part, suggesting a similar role in TC pathophysiology [27]. A hallmark of cancer acknowledged more recently in TC is the avoidance of immune destruction, in which lncRNAs regulate immune checkpoints and shape the tumor immune microenvironment. A recent immune-based molecular classification of PTC has revealed distinct immune-related lncRNA expression profiles associated with immune escape and prognosis [28]. Genome instability and mutation, another hallmark, is facilitated by lncRNAs that alter the repair of cellular DNA. lncRNA SLC26A4-AS1 alters components of the MRN DNA repair complex that facilitate chromosomal instability and ultimately lead to metastasis in thyroid cancer [29]. The final hallmark, tumor-promoting inflammation, is also mediated by lncRNAs. Within thyroid and other endocrine malignancies, lncRNAs have activated NF-κB signaling, establishing a pro-inflammatory microenvironment that promotes tumor growth and spread [30].

Collectively, these findings paint a detailed picture of the role of lncRNAs in affecting the cellular and molecular features of thyroid cancer within the hallmarks of the cancer system. The role of lncRNAs in influencing hallmark features encompasses proliferation, apoptosis, immortality, angiogenesis, invasion, immune evasion, metabolism, genomic instability, and inflammation, indicating that lncRNAs have capacity as both mechanistic drivers of thyroid cancer and valuable clinical targets.

MEG3 is a tumor-suppressive lncRNA that has recently been identified as potentially useful in the clinic in PTC. Using an rRNA-depleted bulk RNA-seq cohort with matched normal tissue and complementary single-cell analyses, observation of the expression of MEG3 was highly associated with lymph node metastasis, and with increased tumor-associated fibrosis characterized by greater infiltration of cancer-associated fibroblasts (CAFs) in human samples. Interestingly, signals indicating MEG3 predominately localize to CAFs in the high-grade subtypes of thyroid cancer, suggesting that changes to the stroma could enable metastatic behavior [31].

The functional studies seem to indicate that MEG3 decreases migration and invasion by decreasing Rac1, through a post-transcriptional interaction with the RAC1 3′UTR. This aligns with its clinical associations and supports its role in the inhibition of metastasis [32]. Overall, these data suggest that MEG3 could function as a context-dependent negative regulator in the tumor microenvironment and a biomarker of nodal spread in PTC [31,32].

#### 3.1.1. Sustaining Proliferative Signaling

Sustained proliferative signaling is a main hallmark of thyroid cancer, due to chronic activation of the main pathways responding to external stimuli, such as MAPK and PI3K/AKT/mTOR. These pathways disregard normal cell growth control to promote tumor evolution or tumor progression. Genetic alterations, in particular, in the receptor tyrosine kinase-RAS-BRAF signaling axis lead to constitutive MAPK pathway activation, and these, in turn, influence tumor differentiation, invasiveness, and microenvironmental alterations [33,34]. The PI3K/AKT/mTOR pathway is also active and can be functionally altered to support cell survival and proliferation, and promote resistance to apoptosis [35]. The MAPK and PI3K/Akt/mTOR signaling complexes also interact with each other and can form a complex web of pathways that adapt and compensate, promoting therapy resistance or allowing tumors to evolve [36]. Other influences are also significant, including thyroid hormone signaling, and work in tandem to enhance proliferative and metabolic reprogramming in cancer cells and support angiogenesis or the Warburg effect in cancerous cells. Thyroid hormones have been demonstrated to coordinate a global metabolic switch by transcriptionally activating, for example, VEGF A and HIF 1α transcripts to facilitate angiogenesis, and concomitantly, upregulating PKM2 and LDHA to promote the production of lactate, even in normoxic conditions, and other features attributed to the Warburg effect [37,38,39]. Targeted therapeutic strategies designed to target the MAPK/PIK3/AKT/mTOR pathways have shown potential; however, resistance remains a critical barrier, supporting the need to develop combination targeting strategies or to target other things [34]. The relationship between these signaling cascades is important for developing better treatment strategies and improving patient outcomes in thyroid cancer [35]. In the context of thyroid cancer, knockdown of LINC00162 in PTC cells has been shown to increase sensitivity to sorafenib through the modulation of MAPK signaling and apoptosis, suggesting that lncRNA–MAPK interactions are crucial for a response to therapy [40].

**Figure 1:**
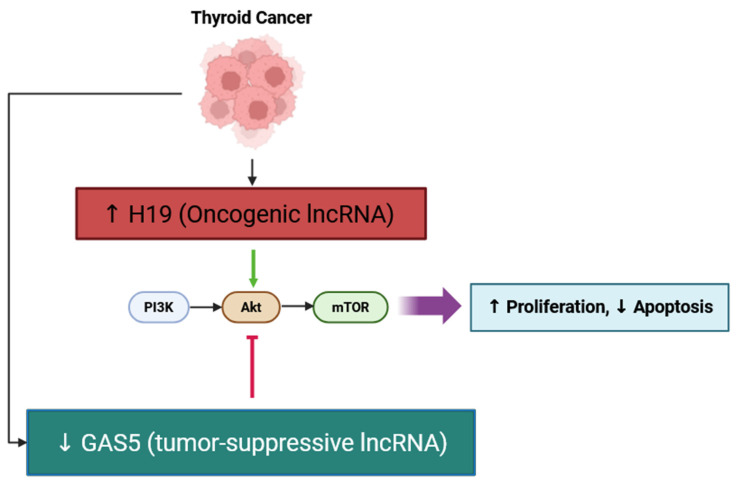
Schematic representation of the regulatory effects of lncRNAs H19 and GAS5 on the PI3K/AKT/mTOR signaling pathway in thyroid cancer. Upregulation of oncogenic lncRNA H19 activates the PI3K/AKT/mTOR cascade, leading to increased proliferation and reduced apoptosis, whereas downregulation of tumor-suppressive lncRNA GAS5 removes inhibitory control over AKT signaling, further enhancing tumor progression. ↑ indicates increase; ↓ indicates decrease; ⊣ represents suppression; Arrow in green color indicates activation.

Long non-coding RNA H19 is upregulated in thyroid cancer tissues and is an important contributor to the promotion of cancer cell proliferation and survival in thyroid cancer cells, at least in part by inducing PI3K/AKT signaling. Studies in thyroid cancer cell lines show that silencing H19 decreased cell viability and increased apoptosis in these cells, and correlated with decreased levels of phosphorylation of PI3K and AKT, suggesting decreased activity of the pathway. This implies that H19 functions as an oncogene in thyroid cancer, enabling the signaling of the PI3K/AKT pathway to promote tumor cell growth and support resistance to cell death. The tumor-suppressive lncRNA GAS5, conversely, inhibits AKT activation, counteracting the pro-tumorigenic effects of H19, as illustrated in Figure 1. In short, targeting H19 or the PI3K/AKT signaling as a therapeutic approach for the management of thyroid cancer is exciting [41]. Mechanisms similar to the role of lncRNA H19 in regulating PI3K/AKT signaling in thyroid cancer have been defined in other endocrine tumors like pancreatic neuroendocrine neoplasms, but in the case of thyroid cancer, it is further evidence that H19 is a central player in tumor progression through the regulation of the PI3K/AKT pathway [42].

#### 3.1.2. Evading Growth Suppressors

**Figure 2:**
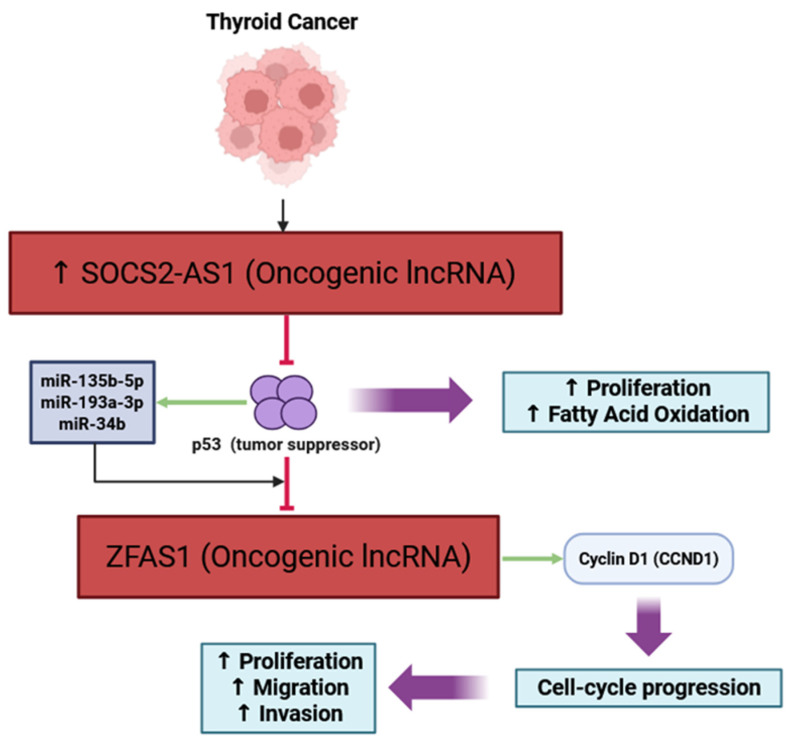
Schematic representation of the involvement of the oncogenic lncRNAs SOCS2-AS1 and ZFAS1 in evading growth suppressors through modulation of the p53 tumor-suppressor pathway in thyroid cancer. SOCS2-AS1 promotes tumor progression by degrading p53 to decrease tumor-suppressive activity and increase proliferation and fatty acid oxidation. Specifically, p53 directly represses ZFAS1 and downregulates it indirectly through p53-induced microRNAs (miR-135b-5p, miR-193a-3p, and miR-34b). If the hallmark activity of p53 is lost, ZFAS1 is upregulated, leading to an increase in cyclin D1 (CCND1) expression, progression through the cell-cycle, proliferation, migration, and invasion. ↑ indicates upregulation; Arrow in green color indicates activation; ⊣ represents inhibitory interaction.

LncRNAs are an important aspect of thyroid cancer biology, disrupting tumor-suppressor signals, including the p53 pathway. In PTC, there is a lncRNA, SOCS2-AS1, that is highly expressed and affects cancer progression by binding and degrading p53, limiting its tumor-suppressive function and promoting cell proliferation and fatty acid oxidation [43]. The oncogenic lncRNA ZFAS1, however, is directly repressed by p53, and regulated by p53 (and its microRNA genes, miR-135b-5p, miR-193a-3p, and miR-34b), so that when these tumor suppressors are present, they inhibit the expression of lncRNA ZFAS1 and thus inhibit PTC cell proliferation [22]. There are two insights to gain from these observations: first, some lncRNAs can inhibit tumor-suppressor pathways by degrading p53; and second, other lncRNAs are inhibited by p53 to prevent tumor growth (Figure 2). Exploring ways to intervene in lncRNA–p53 interactions may provide novel methods to treat thyroid cancer.

Recent studies indicate that the long non-coding RNA ZFAS1 functions as an oncogene in thyroid cancer, with expression upregulated in tumor tissues and cell lines. Knockdown of ZFAS1 led to decreased proliferation, migration, invasion, and EMT in thyroid carcinoma cells, in part by increasing levels of anti-tumor microRNAs such as miR-302a-3p, and subsequently reducing cyclin D1 (CCND1) expression, which promotes cell cycle progression. In medullary thyroid carcinoma, inhibition of ZFAS1 also inhibited tumor growth and metastasis through the miR-214-3p/UCHL1/EPAS1 pathway, providing evidence of its role in cancer aggressiveness [44]. ZFAS1 is repressed by the tumor-suppressor protein p53, and also regulated through p53-induced microRNAs (miR-135b-5p, miR-193a-3p, and miR-34b) that downregulate ZFAS1 and decrease the proliferation of thyroid cancer cells [22]. In summary, ZFAS1 downregulation restored p53-related tumor-suppressor pathways and inhibited proliferation in thyroid cancer by regulating multiple cell cycle regulators and microRNA networks.

#### 3.1.3. Resisting Cell Death

**Figure 3:**
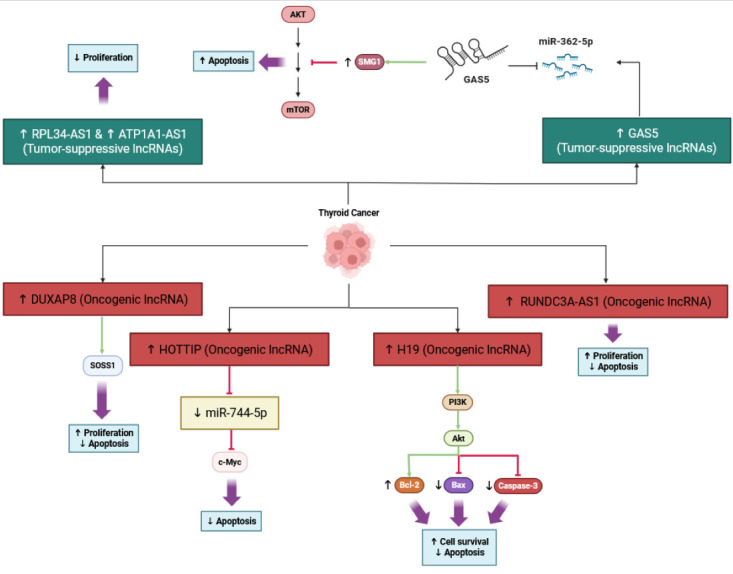
Schematic overview of oncogenic and tumor-suppressive lncRNAs involved in regulating apoptosis resistance in thyroid cancer. Oncogenic lncRNAs such as DUXAP8, HOTTIP, RUNDC3A-AS1, and H19 promote proliferation and inhibit apoptosis through diverse molecular mechanisms, including the modulation of microRNAs (e.g., miR-20b-5p, miR-744-5p, miR-151b) and activation of key signaling pathways (e.g., PI3K/AKT). Conversely, tumor-suppressive lncRNAs, including RPL34-AS1, ATP1A1-AS1, and GAS5, enhance apoptosis and reduce proliferation by regulating target miRNAs and downstream effectors, such as SMG1, leading to inhibition of the Akt/mTOR pathway. These complex interactions highlight the potential of targeting lncRNA-mediated apoptotic pathways as a therapeutic strategy in thyroid cancer. ↑ indicates increase; ↓ indicates decrease; ⊣ represents suppression; Arrow in green color indicates activation.

LncRNAs are important mediators of the resistance to apoptosis in thyroid cancer cells, through interactions with a variety of microRNAs and signaling pathways. For example, DUXAP8 is over-expressed in PTC and promotes resistance to apoptosis. DUXAP8 can enhance SOSS1 and activate downstream proliferative signaling by sponging miR-20b-5p, which is ultimately associated with a poorer prognosis and a higher tumor grade [45]. Similarly, HOTTIP is an oncogene that is also associated with downregulating miR-744-5p, which normally inhibits c-myc and promotes apoptosis; therefore, c-myc downregulation allows for thyroid cancer resistance to apoptosis [46]. Additionally, RUNDC3A-AS1 promotes proliferation in thyroid cancer and inhibits apoptosis via targeting the miR-151b/SNRPB axis [47]. Interestingly, not all lncRNAs are oncogenic; RPL34-AS1 and ATP1A1-AS1 are tumor-suppressor lncRNAs. These over-expressions promote apoptosis, through the modulation of miRNA target expressions and their downstream effectors, thereby inhibiting proliferation [48,49]. Further, lncRNA GAS5 increases sensitivity to radioiodine therapy, following miR-362-5p sponging, promoting SMG1 expression, which then leads to Akt/mTOR inhibition and apoptosis [50]. Collectively, these studies illustrate lncRNA’s critical involvement in complex regulatory networks of apoptosis in thyroid cancer, providing targets for further therapeutic interventions. This regulatory network is illustrated in Figure 3.

The long non-coding RNA H19 regulates the viability of thyroid cancer cells, primarily via the mechanism of regulating the PI3K/AKT survival signaling axis, and the knockdown of H19 has been shown to decrease the viability of thyroid cancer cells at the cell level and to increase apoptosis. H19 is generally upregulated in thyroid cancer tissues, and silencing H19 can increase the expression of the pro-apoptotic proteins, e.g., Bax and caspase-3, and decrease the expression of the anti-apoptotic protein, Bcl-2. In terms of mechanisms, the knockdown of H19 decreased the phosphorylation of PI3K and AKT, consistent with the inhibition of this survival signaling pathway, which is significant for cancer cell proliferation and resistance to cell death. Therefore, there is evidence supporting the conclusion that H19 acts as an oncogene in thyroid cancer, at least in part via activation of the PI3K/AKT pathway, in order to enhance the pro-survival properties of thyroid cancer cells. Ultimately, targeting H19 may represent a novel therapeutic approach to promote apoptosis and inhibit tumor growth in thyroid cancer by inhibiting the activation of the PI3K/AKT signaling axis [41].

#### 3.1.4. Enabling Replicative Immortality

**Figure 4:**
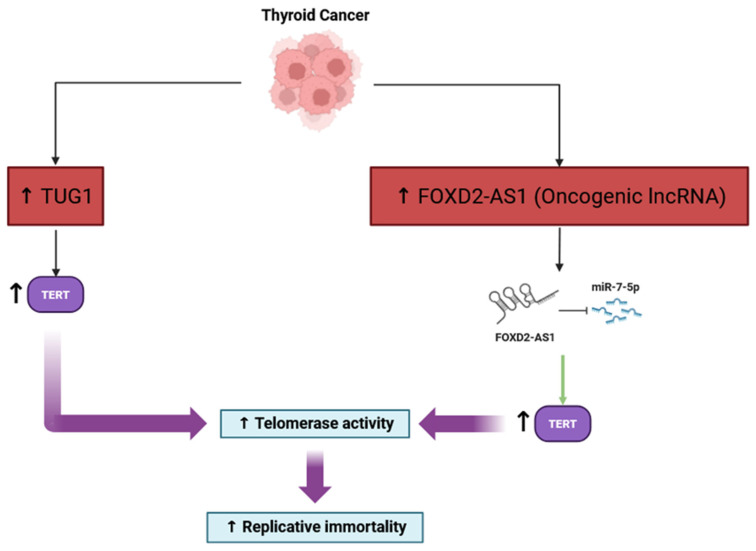
Oncogenic lncRNAs promoting replicative immortality in thyroid cancer through modulation of telomerase activity. Overexpression of TUG1 and FOXD2-AS1 enhances TERT expression, thereby increasing telomerase activity and enabling cancer cells to maintain telomere length during continuous cell division. FOXD2-AS1 also acts by sponging miR-7-5p, which normally suppresses TERT, further contributing to sustained telomerase activity. These mechanisms collectively facilitate replicative immortality, a hallmark of thyroid cancer progression. ↑ indicates increase; Arrow in green color indicates activation.

LncRNAs are important players in regulating replicative immortality in thyroid cancer. They impact telomerase activity, which maintains telomere length during continuous rounds of cell division in cancer. In thyroid cancers, particularly the papillary and anaplastic forms, lncRNAs can modulate the expression and activity of TERT, the catalytic subunit of telomerase, through various mechanisms such as epigenetic regulation, sponging of microRNAs, and cooperating with signaling pathways such as PI3K/Akt and Wnt [51]. These regulatory activities can lead to an increased abundance of TERT, which leads to increased telomerase activity in thyroid cancer cells, allowing cancer cells to avoid senescence and lapse into replicative immortality [52] (Figure 4). Furthermore, lncRNAs may also influence TERT promoter methylation, histone modifications, and alternative splicing, adding another layer of control to telomerase functions in thyroid cancer cells [53]. A number of lncRNAs have been identified as candidate diagnostic or prognostic biomarkers, and the dysregulation of lncRNAs has been associated with tumor progression and aggressiveness [54]. That being said, it is still unclear which lncRNAs are relevant to thyroid cancer, and the full descriptions of their molecular actions in thyroid cancer are still under investigation. This suggests that there is a substantial opportunity for future research to discover novel treatment, as the targets have significant implications for improving clinical outcomes [55].

LncRNAs are pivotal in modulating gene expression and cellular processes that further the immortalization and progression of TC cells. Partially realized, this provides confronting evidence of lncRNA modulating TERT, whereby TERT itself has been demonstrated to be regulated by spacial compartmentalization, intron retention, and eventually TUG1, that may influence cell viability and be manipulated for therapy in the RNA space when targeting the telomerase activity central to cellular immortalization [56]. In particular, regarding TC, THER-saturated expression of the lncRNAs MALAT1 and ACVR2B-AS1 enhanced proliferation, migration, and invasion of cancer cells, by acting as microRNA sponges that would upregulate oncogenic targets and pathways, with TERT’s direct involvement still obscured. These cells are common and typically found to be overexpressed in TC; for that matter, they are particularly associated with poor prognosis, and sustaining the malignant phenotype, possibly supporting telomerase activity indirectly [57,58]. Nevertheless, lncRNA are attractive biomarkers and therapeutic candidates that function in controlling or regulating gene expression networks, including those linked to cell immortality.

Recent studies indicate that TERT promoter mutations are a prominent mechanism of telomerase activation in thyroid cancer, correlating with enhanced TERT expression and increased aggressiveness [59]. While lncRNA dysregulation has been associated with thyroid cancer progression and telomerase regulation, limited data are currently available in the literature that relate specific changes in lncRNA expression to TERT promoter mutations. Most studies relate to the alterations in the genetic and epigenetic mechanisms regulating TERT expression and telomerase activation [53], including those associated with TERT promoter mutations, interactions, DNA methylation, and histone modifications. There are some reports estimating that TERT can even be upregulated in the absence of the TERT promoter mutation, possibly through other pathways and not changing the expression of lncRNA [60]. Although both lncRNA dysregulation and TERT promoter mutations are significant features in thyroid cancer biology and telomerase dysregulation, the studies cited herein do not provide evidence that directly associates changes in lncRNA with TERT promoter mutations in thyroid cancer.

#### 3.1.5. Inducing Angiogenesis

**Figure 5:**
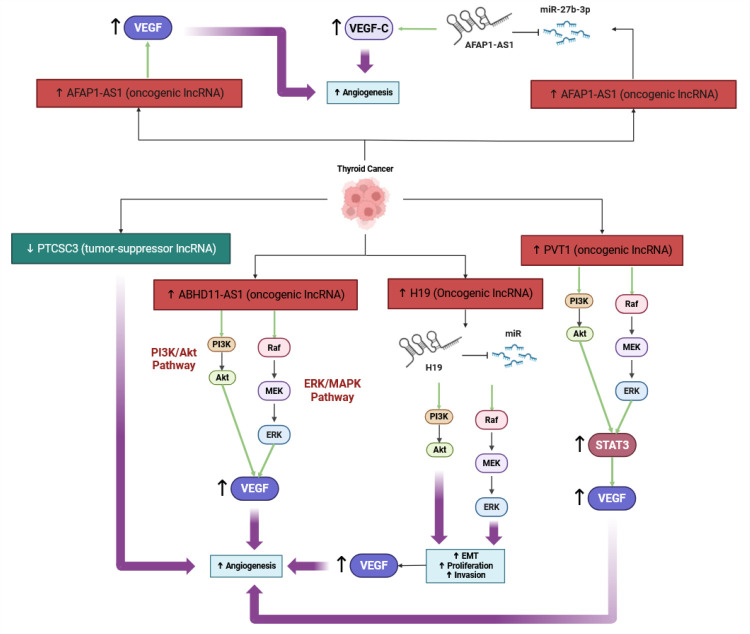
Graphical summary of lncRNA-mediated regulation of angiogenesis in thyroid cancer. Oncogenic lncRNAs (H19, ABHD11-AS1, PVT1, and AFAP1-AS1) upregulate VEGF expression and angiogenesis through several mechanisms, including sponging microRNAs’ (miR-27b-3p) activity to lower their concentrations, activating PI3K/Akt signaling and ERK/MAPK signaling, and stabilizing STAT3. Tumor-suppressor lncRNAs (e.g., PTCSC3) are downregulated to promote enhanced vascularization. Overall, these factors promote EMT, proliferation, and invasion, and the establishment of a pro-angiogenic tumor microenvironment. ↑ indicates increase; ↓ indicates decrease; ⊣ represents suppression; Arrow in green color indicates activation.

LncRNAs are likely critical regulators of tumor angiogenesis, influencing gene expression, tumor growth, and vascularization pathways in thyroid cancer. Some lncRNAs, including H19 and ABHD11-AS1, are upregulated in thyroid tumors, which enhance EMT, proliferation, invasion, and angiogenesis, most often by acting as sponge molecules for microRNAs, or modulating key pathways, such as PI3K/Akt and ERK/MAPK [61]. For instance, H19 can promote angiogenesis and tumor growth, although it can have differing roles depending on the cancer context [62]. While some lncRNAs, such as PTCSC3, act to suppress tumors, their downregulation correlates to an increase in angiogenesis and aggressiveness of the tumor [63]. In addition to their interactions with microRNAs, lncRNAs also likely affect the tumor microenvironment, contributing to the development of new blood vessels and metastasis [64] (Figure 5). As lncRNAs are identified as prognostic biomarkers or a target for existing therapies in thyroid cancer, negative regulators of angiogenesis may be identified that will limit tumor progression by blocking angiogenesis.

LncRNAs have been identified as promoting the upregulation of vascular endothelial growth factor (VEGF) expression, and potentially increasing the levels of angiogenesis in various cancers, including TC and other solid tumors. For example, lncRNA PVT1 promotes angiogenesis by stabilizing STAT3, which then activates the STAT3/VEGFA signaling axis that drives upregulation of VEGF expression and tumor progression in gastric cancer—this is likely relevant to other cancers as well [65]. LncRNA ANRIL likewise upregulates VEGF and promotes angiogenesis through the activation of the NF-κB signaling pathway, as was shown in a rat model of diabetes mellitus and cerebral infarction [66]. LncRNA AFAP1-AS1 regulates VEGF-C by sponging miR-27b-3p in cervical cancer, to maintain cancer stem cell properties and angiogenesis [67]. Furthermore, lncRNA SNHG1 also modulates the HIF-1α/VEGF pathway as a competing endogenous RNA, which helps enhance endothelial cell proliferation and tube formation under hypoxic conditions [68]. Environmental exposure to PM2.5 can also trigger lncRNA-mediated VEGF upregulation, as seen with lncRNA NONHSAT021963 in lung cancer cells to further promote angiogenesis [69]. Collectively, these studies suggest that lncRNAs have diverse multi-faceted ways in which they may upregulate VEGF expression and facilitate angiogenesis and offer potential therapeutic targets for disrupting tumors and mediating vasculature, reducing tumor vascularization and proliferation.

#### 3.1.6. Activating Invasion and Metastasis

**Figure 6:**
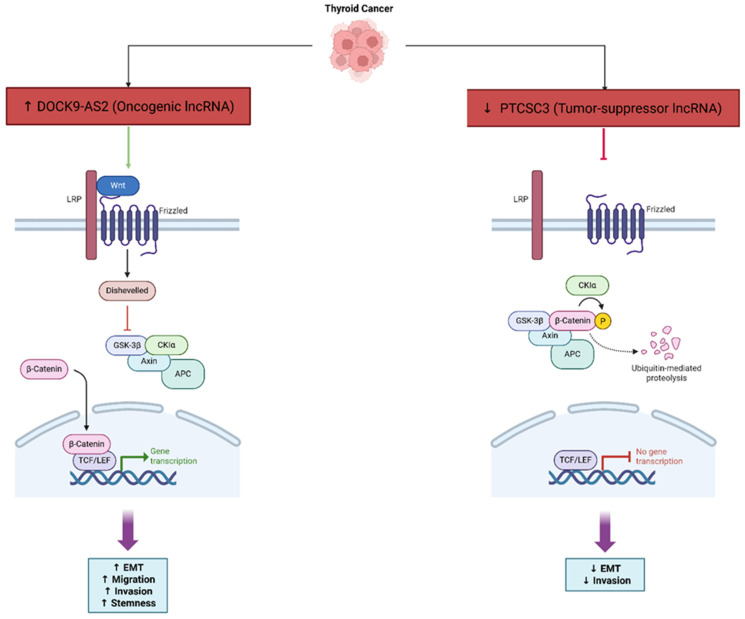
Schematic illustration of lncRNA-mediated regulation of Wnt/β-catenin signaling in thyroid cancer. Oncogenic lncRNA DOCK9-AS2 activates β-catenin transcriptional activity while promoting EMT, migration, invasion, and stemness. Tumor-suppressor lncRNA PTCSC3 inhibited Wnt/β-catenin signaling leading to decreased EMT and invasion. ↑ indicates increase; ↓ indicates decrease; ⊣ represents suppression; Arrow in green color indicates activation.

LncRNAs are involved in regulating EMT and invasion in thyroid carcinoma, especially in PTC. LncRNAs can act as promoters or suppressors of tumor development by regulating important signaling pathways, including PI3K/Akt and Wnt/β-catenin. A common method by which lncRNAs can control gene expression is through microRNA sponging and transcription regulation [51]. LncRNA PTCSC3, a tumor suppressor, suppresses EMT and invasion via the Wnt/β-catenin signaling pathway, and the downregulation of PTCSC3 is associated with a higher risk of cancer and aggressiveness [63] (Figure 6). Studies have shown that there are several other lncRNAs that can influence EMT and invasion, such as BANCR, though their role in thyroid cancer is still not clear because studies have produced conflicting evidence [70]. The presence of the ceRNA network, containing lncRNAs, microRNAs, and other non-coding RNAs, makes it even more complex, because lncRNA, microRNA, and their interactions can promote metastasis, migration, and drug resistance in thyroid cancer [71]. In addition, lncRNAs impact the tumor immune microenvironment and processes such as recruitment and polarization of immune cells, which directly influences EMT and invasion [72]. Overall, the understanding of lncRNA regulatory mechanisms ultimately enhances our understanding of thyroid cancer biology, but they could also serve as potential diagnostic biomarkers and novel therapeutic targets. PICSAR was revealed as a pivotal oncogenic lncRNA that may stimulate invasion and proliferation of thyroid cancer cells through the miR-320a/miR-485–RAPGEFL1 axis, among oncogenic lncRNAs, which emphasized the role of ceRNA-mediated signaling in the metastatic phase [73].

LncRNA DOCK9-AS2 is a critical contributor to stemness and invasiveness in PTC. It is found to be upregulated in PTC tissues, and is also particularly found in exosomes shed from cancer stem cell-like cells. Functional evidence shows that DOCK9-AS2 knockdown reduces PTC cell proliferation, migration, invasion, EMT, and stemness. Mechanistically, DOCK9-AS2 binds to transcription factor SP1, causing increased expression of catenin beta 1 (CTNNB1), and acts as a molecular sponge for miR-1972, which upregulates CTNNB1. Hence, DOCK9-AS2 ultimately activates the Wnt/β-catenin signaling pathway to induce cancer progression and stemness. To further support this, the exosomal DOCK9-AS2 transfer from cancer stem cells to other PTC cells provides more stemness. This suggests that DOCK9-AS2 also plays a role in the aggressiveness of tumors and possibly treatment resistance. Overall, these findings indicate that DOCK9-AS2 is a viable therapeutic target to limit the progression of PTC and patient outcomes [74].

There is strong evidence of crosstalk between lncRNAs and TGF-β signaling in thyroid cancer, especially for the regulation of EMT. For example, the lncRNA FOXD3-AS1 has been demonstrated to promote aggressive traits in thyroid cancer by altering the TGF-β1/Smads pathway; FOXD3-AS1 depletion inhibits EMT and tumor progression through inactivation of the TGF-β signaling pathway by upregulation of miR-296-5p [75]. Similarly, the lncRNA CATIP-AS1 modulates EMT in thyroid cancer cells through a regulatory axis involving miR-515-5p and the TGF-β signaling mediator Smad4 [76]. There are reviews that also summarize that lncRNAs can either positively regulate TGF-β signaling, or be negatively regulated, which can influence cancer cell plasticity, invasion, and metastasis by TGF-β [77]. The evidence of crosstalk between lncRNAs and TGF-β signaling is likely an important mechanism by which lncRNAs promote thyroid cancer progression and may be a viable target for intervention [78].

#### 3.1.7. Deregulating Cellular Energetics

LncRNAs are highly involved in the metabolic reprogramming of thyroid cancer and impact tumor growth, progression, and stress adaptation. In PTC, metabolic plasticity comprises aerobic glycolysis, mitochondrial remodeling, and redox control. Sirtuines (SIRT1/SIRT3) are NAD^+^-dependent deacetylases that integrate metabolic status with corresponding transcriptional programs of glycolysis and oxidative phosphorylation. Clinically, high SIRT1 corresponds negatively with lymphovascular invasion and both central and lateral lymph node metastasis, and high SIRT3 correlates positively with locoregional recurrence; together, low SIRT1/high SIRT3 stratify patients with more aggressive phenotypes, highlighting both a prognostic dichotomy and direct correlation with metabolic phenotypes in PTC [79]. In agreement with this finding, research across cancer types supports lncRNA→miRNA→sirtuin pathways that connect non-coding RNA signaling with metabolic regulation: in particular, a validated MEG3–miR-181a–SIRT1 axis is associated with markers of metabolic reprogramming in human cohorts and illustrates how lncRNAs can influence sirtuin activity indirectly [80]. Although thyroid-specific upstream lncRNAs for SIRT1/3 have yet to be completely elucidated, the overlap between sirtuin biology and lncRNA/miRNA networks is suggestive of testable mechanisms by which lncRNAs could enforce glycolytic dependence or relieve glycolytic dependence in PTCs [79].

**Figure 7:**
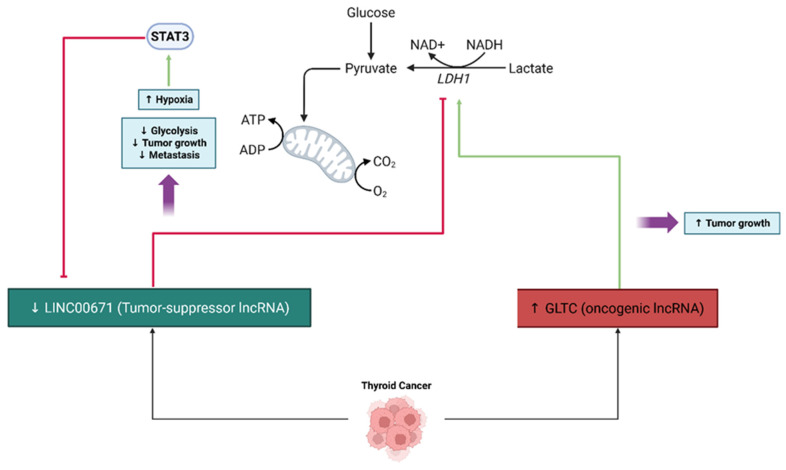
LncRNA-mediated regulation of metabolic reprogramming in thyroid cancer. Downregulation of tumor-suppressor lncRNA LINC00671 increases STAT3 activity and glycolysis, driving tumor growth and metastasis. In contrast, lncRNA GLTC (oncogenic lncRNA) enhances LDH1-dependent lactate production and stimulates tumor growth through glycolytic reprogramming. ↑ indicates increase; ↓ indicates decrease; ⊣ represents suppression; Arrow in green color indicates activation.

In PTC, lncRNAs modulate important signaling pathways, such as PI3K/Akt and Wnt, which are related to metabolic change and the survival of cancer cells, often by acting as microRNA molecular sponges and regulating the expression of genes related to metabolic processes and the epithelial–mesenchymal transition [51]. In ATC, the research on lncRNAs is not as established, yet several lncRNAs have been identified as regulators of tumorigenesis and potential biomarkers or therapeutic targets, with some governing metabolic pathways via epigenetic mechanisms or post-transcriptional regulation [54]. Generally, lncRNAs are recognized as fine-tuning regulators of gene expression, and regulate protein synthesis and metabolic adaptation for nutrient and oxygen restriction for cancer cells by prioritizing the translation of specific oncogenes and metabolic enzymes [81] (Figure 7). Certain lncRNAs, including ABHD11-AS1, are upregulated in thyroid cancer and participate in oncogenic processes by altering pathways and epigenetic states that would lead to metabolic reprogramming [82]. LncRNAs can also bind to major metabolic regulatory proteins, such as MYC, and form feedback loops that increase glycolysis and other metabolic shifts associated with cancer biomass [83]. Overall, these results reveal the important role lncRNAs play in modulating the metabolic flexibility of thyroid cancer, which implies the potential of lncRNAs to be diagnostic markers and/or therapeutic targets.

LncRNAs are greatly associated with regulating glycolytic metabolism to provide energy for rapidly proliferating thyroid cancer cells. For instance, LINC00671 is a potential tumor suppressor, as it downregulates lactate dehydrogenase A (LDHA), a major glycolytic enzyme, restraining glycolysis, tumor growth, and metastasis, before its expression is inhibited under hypoxic conditions through the activation of STAT3, which enhances LDHA expression and enhances cancer progression [84]. Conversely, the lncRNA GLTC, which is upregulated in papillary thyroid cancer, interacts with LDHA to increase its succinylation and activity, which promotes glycolysis, and the tumor growth was induced during treatment due to resistance to radioiodine therapy, as shown through subsequent experiments [85]. The findings, coupled with the previously established roles for lncRNAs in modulating metabolism, illustrate the potential application of targeting lncRNAs or downstream pathways for the therapeutic index, and persistence of thyroid cancer progression and treatment resistance.

#### 3.1.8. Avoiding Immune Destruction

**Figure 8:**
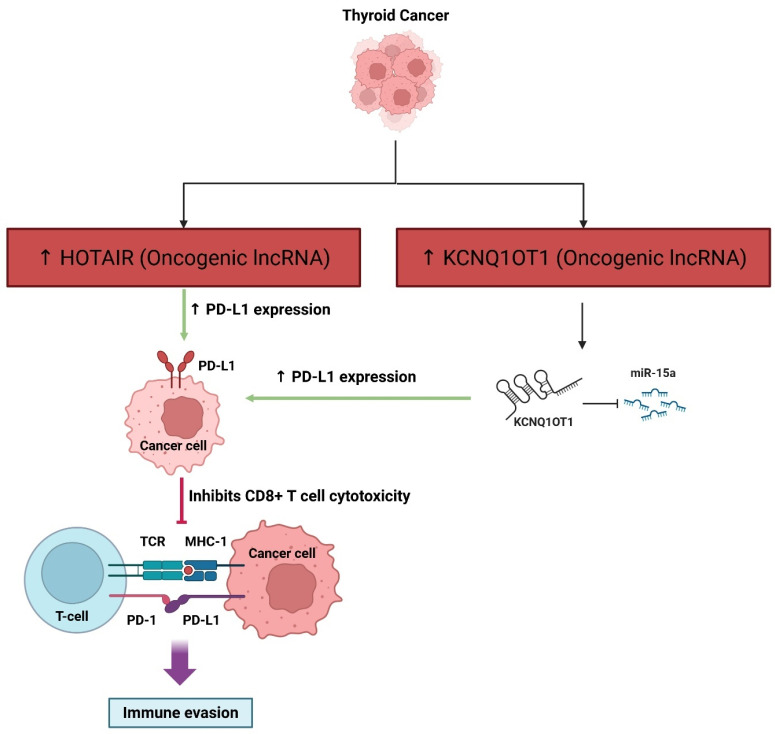
LncRNA-mediated immune evasion mechanisms in thyroid cancer. Oncogenic lncRNAs such as HOTAIR and KCNQ1OT1 induce PD-L1 expression, either directly or by sponging miR-15a to inhibit CD8+ T cell cytotoxicity and enhance immune evasion. Such mechanisms drive immune suppression within the tumor microenvironment and resistance to therapies and are known to support immune evasion mechanisms in thyroid cancer. ↑ indicates increase; ⊣ represents suppression; Arrow in green color indicates activation.

LncRNAs are significant for regulating immune evasion and shaping the TME in thyroid cancer and other cancers. These molecules regulate immune cell activities, such as macrophage polarization, T cell activity, and MDSCs, by regulating gene expression, cytokine signaling, and cellular processes in the TME, thereby subverting anti-tumor immunity and promoting expansion, immune evasion, and therapy resistance [86]. LncRNAs function as scaffolds, decoys, and sponges and modulate both innate and adaptive immune responses, and are frequently packaged in exosomes for communication between the tumor and the immune cells [87]. Notably, lncRNAs mediate the recruitment of tumor-associated macrophages and M2 polarization, which support tumor growth and mediate anti-tumor immunity suppression [88]. In thyroid cancer, the immune microenvironment is a highly complex and heterogeneous state, and lncRNA-mediated modulation of immune cells could well contribute to the heterogeneity of outcomes for various immunotherapies [89]. Targeting lncRNAs may be an opportunity to enhance pathogenic immunity, create drug resistance, and to act as prognostic biomarkers or therapeutic targets [90]. However, despite promising results, the signaling mechanisms in which lncRNA mediates immune evasion in thyroid cancer require additional study and exploration, in order to translate findings to the clinical context (Figure 8).

LncRNAs are important to tumor immune evasion in many cancers, including TC and solid tumors. For instance, the lncRNA named NKILA can induce immune evasion by increasing the susceptibility of CTLs and type one helper T cells to activation-induced cell death, thus limiting effective anti-tumor immune responses and becoming associated with poor survival outcomes [91]. Other lncRNAs, such as HOTAIR, can also be delivered in extracellular vesicles and promote immune evasion by increasing the expression of PD-L1, which can impair T cell-mediated cytotoxicity and facilitate tumor resistance to chemotherapy [92]. KCNQ1OT1 behaves as a molecular sponge for miR-15a, increasing PD-L1 expression and inhibiting CD8+ T cell cytotoxicity, leading to immune evasion and tumor progression [93]. Moreover, MIAT in hepatocellular carcinoma is linked to increased expression of immune checkpoint molecules (PD-1, PD-L1, CTLA4) and found in populations of immune cells associated with immune suppression. This implies that MIAT is involved in the tumor’s immune microenvironment and drug response [94]. In summary, lncRNAs contribute to immune evasion during tumor development by regulating immune checkpoints, and modulating T cell function and changes in the tumor microenvironment, making them good targets for developing new immunotherapeutic approaches.

#### 3.1.9. Genome Instability and Mutation

LncRNAs are important factors in the genomic instability and mutations that are present in thyroid cancer, especially by modulating gene expression, mediating epigenetic changes, and defining the activity of the microRNAs and signaling pathways that ultimately lead to tumorgenesis and cancer progression [95]. In thyroid cancers, such as papillary or follicular, or less commonly in the more aggressive anaplastic type, lncRNAs can modulate gene expression either as oncogenic or tumor-suppressive types, potentially affecting biological processes such as cell proliferation, apoptosis, the epithelial–mesenchymal transition, and metastasis [51]. LncRNAs like PTCSC3 mainly exert their function as a tumor suppressor, and its downregulation can place individuals at a higher risk for developing malignancy and/or concerning genomic instability. Other lncRNAs, such as ABHD11-AS1, are associated with poor prognosis and oncogenic activity [63]. By modulating important biological processes such as DNA repair, chromatin structure, and gene expression involved with maintaining genome stability, lncRNAs have the capacity to induce genomic instability [96]. Furthermore, lncRNAs are actively involved in ceRNA networks, and can sequester microRNAs to indirectly modulate the expression of genes that are critical to DNA stability and the mutation rate [71]. Furthermore, the dysregulation of specific lncRNAs has been seen in thyroid cancer tissues and is associated with the accumulation of genetic and epigenetic alterations that ultimately promote tumor progression and reduce the response to treatment [54]. The identification and characterization of lncRNAs in thyroid cancer not only improves the understanding of the mechanisms of genomic instability but also provides the opportunity for new biomarkers for diagnosis, prognostic prediction, and targeted therapeutic approaches [82]. However, more studies will need to be performed to provide a complete picture of the functional roles of many lncRNAs and their relationship with genomic instability and mutation in thyroid cancer [55].

Dysregulated lncRNAs in thyroid cancer can alter mutation rates and chromosomal instability: for example, in SLC26A4-AS1. SLC26A4-AS1 had a downregulated gene expression in thyroid cancer, and its loss is associated with increased DNA double-strand break repair by the MRN complex. The MRN complex can drive genomic instability and metastatic potential by the upregulation of DNA repair signaling pathways to promote the survival and proliferation of cancer cells, despite accumulating unrepaired DNA damage [29]. Dysregulated lncRNAs are sometimes attenuated in copy number variations at the lncRNA loci in thyroid cancer. This suggests that genomic instability may be a driver, as well as a consequence, of the copy number variations present in lncRNA [97]. Additional lncRNAs, where lncRNA dysregulation is loss of function with respect to SOCS2-AS1, the lncRNA can disrupt the function of the tumor suppressor p53, therefore compromising the integrity of the genome and resulting in higher rates of proliferation among cancer cells [43]. By examining the genome-wide studies performed, 100s of lncRNAs show upregulated or downregulated expression, which correlates to a worse prognosis, lymph node metastasis status, and mutations in significant oncogenes (like BRAF(V600E), which is also tied to higher chromosomal instability [97,98]. Although this needs to be shown for any lncRNAs in thyroid cancer, lncRNA dysregulation also influences DNA damage-repair kinetics, as well as chromosomal stability or instability. Given both, there is the potential for downstream consequences of mutation, and alteration in expression of specific lncRNAs may lend themselves towards targeting to mitigate mutation frequency and ultimately, patient outcomes.

New evidence demonstrates an association of altered lncRNA expression to the presence of oncogenic mutations, such as BRAF and RET, in thyroid follicular cell-derived cancers. For instance, the lncRNA CASTL1 has been found to promote the CCDC6-RET fusion (RET/PTC1) that is frequently expressed in papillary thyroid carcinoma by facilitating chromosomal rearrangements; in tumors with the CCDC6-RET fusion, there is increased CASTL1 expression, demonstrating a potential mechanism for how lncRNA dysregulation might lead to formation of oncogenic mutations [99]. In addition, the lncRNA FAM111A-DT is also upregulated in papillary thyroid cancer and has been linked to BRAF^V600E oncogenic mutation, suggesting that specific lncRNAs may be linked with a specific oncogenic mutation, which may be correlated with more aggressive tumor behavior [100].

Reviews have also supported the idea that lncRNAs may play important roles in thyroid cancer progression, in regulating the expression of genes, proliferation, apoptosis, and metastasis; lncRNAs are consistently noted to have altered expression in tumors with known oncogenic mutations [95]. Although these findings support a connection between altered lncRNA expression and oncogenic mutations in follicular-derived thyroid cancer, the underlying regulatory network and causative relationship between the alterations in lncRNA expression and the development of a specific oncogenic mutation still needs to be more adequately understood [95,99,100].

#### 3.1.10. Tumor-Promoting Inflammation

**Figure 9:**
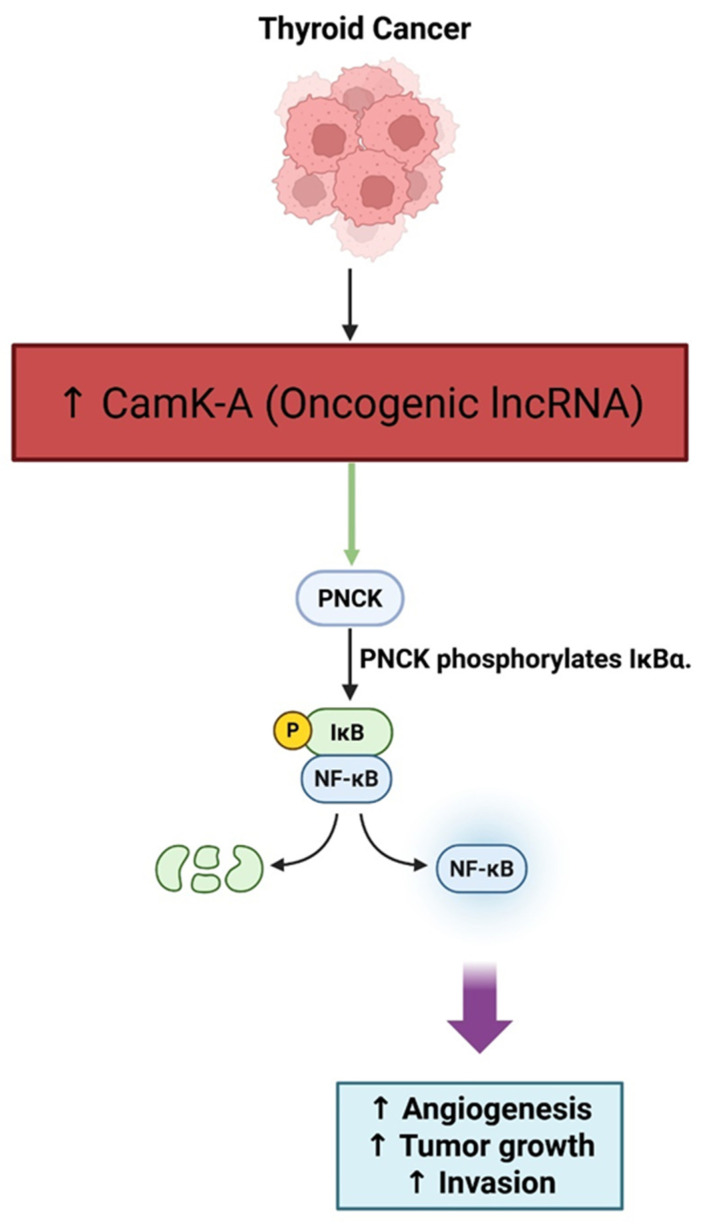
Oncogenic lncRNA CamK-A activates tumor-associated inflammation in thyroid cancer. CamK-A activates PNCK, which then phosphorylates IκBα, leading to NF-κB activation. Once activated, NF-κB mediates angiogenesis, tumor growth, and invasion by modulating the tumor microenvironment and promoting inflammatory signaling. ↑ indicates increase; Arrow in green color indicates activation.

LncRNAs are implicated in promoting tumor-associated inflammation in thyroid carcinoma through the modulation of the tumor microenvironment and immune responses. In the case of papillary thyroid carcinoma, lncRNAs are involved in the regulation of multiple important signaling pathways, such as PI3K/Akt and Wnt and the facilitation of the epithelial–mesenchymal transition, and they act as molecular sponges for microRNAs: all of which promote tumor progression and immune evasion [51]. Exosomal lncRNAs, which are secreted from tumor cells, facilitate communication between cancer and immune cells, which often results in suppression of anti-tumor immunity and therapeutic resistance [101]. These lncRNAs can drive the recruitment and polarization of tumor-associated macrophages (TAMs) toward a pro-tumorigenic and inflammatory M2 phenotype, therefore enhancing inflammation and supporting tumorigenesis [87]. Furthermore, lncRNAs can also impact immune cell plasticity influencing macrophages, T cells, and myeloid-derived suppressor cells, as well as impacting the secretion of inflammatory cytokines such as TNF and IL-6, which also provide an environment that is conducive for tumor progression and inflammation [86]. This is achieved through interfering with antigen presentation and immune surveillance, allowing the thyroid cancer cells to escape detection by the immune system and providing further resistance to immunotherapy [102]. In conclusion, the potential of targeting lncRNA-mediated pathways to interrupt tumor-promoting inflammation and improve therapy in this context is encouraging (Figure 9).

LncRNAs have become recognized as significant modulators of the tumor microenvironment and inflammatory signaling in many cancers, including thyroid cancer. One of the more notable instances is lncRNA CamK-A, which is expressed at high levels in many human cancers, and promotes tumor progression via calcium-dependent NF-κB signaling. CamK-A is able to do so by activating the Ca^2+^/calmodulin-dependent kinase PNCK, leading to the phosphorylation of IκBα, which activates NF-κB, which remodels the tumor microenvironment in several ways by promoting macrophage recruitment, angiogenesis, and tumor growth. The high expression levels of CamK-A were shown to be associated with poor patient survival, suggesting that CamK-A could represent a valuable prognostic biomarker and target for therapeutic intervention. While there have been few direct studies of CamK-A in thyroid cancer, NF-κB signaling is known to be constitutively activated in aggressive thyroid cancers, particularly those with BRAFV600E mutations, and NB-κB signaling has been shown to have increased invasive properties and poor prognosis. There is also evidence for another kinase NIK to be a major driver of NF-κB mediated in thyroid cancer, further establishing the role for NF-κB signaling in promoting a pro-tumorigenic inflammatory milieu. Given all of this feasibility, lncRNAs that can activate NF-κB, like CamK-A, could support the inflammatory and metastatic behavior of thyroid tumors and be feasible targets for future therapy [103,104].

### 3.2. Clinical Implications of lncRNA Dysregulation in Thyroid Cancer

There is significant evidence that an altered expression of lncRNAs influences the clinical course of PTC, and many studies have assessed its potential prognostic value. Multiple lncRNAs including FAM111A-DT [100], LINC02407 [105], SOX2OT, DANCR [106], and others have been shown to correlate with poor prognosis, aggressive tumor behavior, lymph node metastasis, and other unfavorable clinicopathologic parameters in PTC patients, supporting their potential as prognostic biomarkers. In addition, lncRNA-based prognostic risk models have been developed and validated that show very high predictive power for patient outcomes and classify patients into high- and low-risk groups [107]. Importantly, several studies have analyzed lncRNA expression in fine-needle aspiration (FNA) samples and have demonstrated that lncRNA panels can differentiate malignant thyroid nodules from benign thyroid nodules with high sensitivity and specificity, and that they may add to the diagnostic accuracy of indeterminate cytology [108]. Altogether, these findings support the feasibility of lncRNA profiling in FNA samples and suggest incorporation into clinical practice for both the diagnosis and prognosis of PTC, though large-scale validation studies are needed.

Beyond thyroid cancer, siRNA-mediated downregulation of oncogenic lncRNAs such as DLGAP1-AS2 has been shown to improve carboplatin chemosensitivity in lung cancer cells, reinforcing the translational promise of ncRNA modulation as a therapeutic paradigm [109].

A variety of original studies have investigated the functional consequences of specific lncRNAs in thyroid cancer, mapped onto the framework of cancer hallmarks (Table 1). LncRNAs have emerged as beneficial diagnostic and prognostic biomarkers in thyroid cancer, specifically PTC. Numerous studies have reported specific lncRNAs, including lnc-MPEG1-1:1, LINC02407, LINC02471, DOCK9-DT, and LINC00657, as being expressed differently in thyroid cancer tissues than in normal tissues, along with expression levels that correlate with tumor development, lymph node metastasis, and patient survival outcomes [105]. Evidence from diagnostic models and risk signatures based on panels of lncRNAs, including nine lncRNAs and seven lncRNAs, have shown better sensitivity and specificity for distinguishing PTC and predicting prognosis than just using clinicopathological features, with nearly half of the AUCs exceeding 0.8 [107,110]. Some lncRNAs, such as LINC00657, and SLC26A4-AS1, are associated with polarized immune cell types that may also affect the tumor microenvironment and may suggest drug and/or immunotherapy responses [111,112]. There are circulating lncRNAs such as GAS8-AS1 that are emerging as a blood-based, non-invasive diagnostic biomarker in diagnosing thyroid cancer [113]. Functional analyses indicate that these lncRNAs are involved in important cancer-relevant pathways, primarily involving cell proliferation and migration, cell adhesion, and immune regulation, likely through various ceRNA networks with miRNAs and mRNAs [114]. In conclusion, lncRNAs have the potential to broaden the horizons of thyroid cancer diagnosis and prognosis, and to improve personalized therapy approaches.

Therapeutic approaches involving lncRNAs in thyroid cancer are in development, with options like CRISPR/Cas9, antisense oligonucleotides, and RNA interference demonstrating the potential to modulate lncRNA function. These methods are aimed at either inhibiting oncogenic lncRNAs, or restoring tumor-suppressive lncRNAs, which in turn affect the proliferation, invasion, metastasis, and therapeutic resistance of cancer cells. For instance, silencing oncogenic lncRNAs such as GLTC or FAM230B may inhibit tumor progression and diminish radioiodine resistance, and, in contrast, restoring tumor-suppressive lncRNAs such as SLC26A4-AS1, SPTY2D1-AS1, LINC00969, or GAS8-AS1 could also inhibit metastasis and enhance chemosensitivity or autophagy in thyroid cancer cells [29,85,130,131,132,133]. ASOs and RNAi allow for the specific degradation or blockage of lncRNAs, providing evidence from human preclinical models of knockdown of FOXD3-AS1 or MIAT that led to the suppression of aggressive cancer phenotypes and modified key signaling pathways [19,75]. CRISPR/Cas9 provides the ability for permanent changes in gene editing, permitting specific targeting and effects of either disrupting lncRNA loci or the activation of lncRNA loci, although it is still largely experimental in thyroid cancer. These approaches are further supported by research that shows that lncRNAs regulate pathways that are critical in the biology of cancer, including pathways mainly involving aspects of DNA repair, epithelial–mesenchymal transition, and drug resistance, by acting as molecular sponges for microRNAs or by binding to proteins [134]. In summary, the use of promising molecular tools to target lncRNAs provides a substantial opportunity for novel, individualized therapies for thyroid cancer, but additional clinical validation is still required. Nonetheless, the therapeutic application of small ncRNAs still faces practical barriers, especially in delivery and stability. Biodegradable nano-polymers, including chitosan, PLGA, and hyaluronic acid-based systems, are now being developed to act as efficient and safe carriers for antisense oligonucleotides and siRNAs to address the major delivery-related shortcomings of ncRNA-based therapies [135].

There are several important elements and challenges that need to be addressed when considering lncRNA research in the clinical space for thyroid cancer. Several circulating lncRNAs, like GAS8-AS1 and ABHD11-AS1, have promise as targets for diagnostic and prognostic biomarkers, with reasonable specificity and sensitivity; however, their clinical applicability is compromised by variability in results that are day-to-day or between studies, along with limited validation across diverse patient populations [113]. While the functional implications of lncRNAs in thyroid cancer, including their role in major signaling pathways and in the process of the epithelial–mesenchymal transition is quickly evolving, the data concerning individual lncRNAs in thyroid cancer is typically not as consistent or comprehensive as desired, especially for more aggressive and advanced subtypes like anaplastic thyroid cancer [51]. Up to the present moment, the majority of studies have been either preclinical (i.e., mostly combinations of lncRNA with other biomarkers, or more commonly, cell line studies) or based on small cohorts of previously treated patients with not many lncRNAs, robustly linked to prognostic or therapeutic responses [54]. Periodically, lncRNA therapeutics have not even progressed beyond preclinical science, or are just entering an early phase (and, in most cases, none are approved for thyroid cancer, e.g., antisense oligonucleotides, RNA mimics, etc.) [136]. The technical barriers are maintaining the agent stability, delivery, and targeting effectiveness, as well as achieving standardization in detection methods for clinical implementation [113]. Concurrently, it is necessary to understand the off-target effects and long-term safety of lncRNA manipulation in patients [136]. Overall, lncRNAs have great potential for improving thyroid cancer diagnostics, prognostics, and therapy, but larger-scale and well-designed studies and technologic advances are needed before a reliable introduction into the clinic. Key dysregulated lncRNAs with clinical implications, including their roles as biomarkers and therapeutic targets, are summarized in Table 2.

### 3.3. lncRNA–miRNA Crosstalk in Cancer Immunity

**Figure 10:**
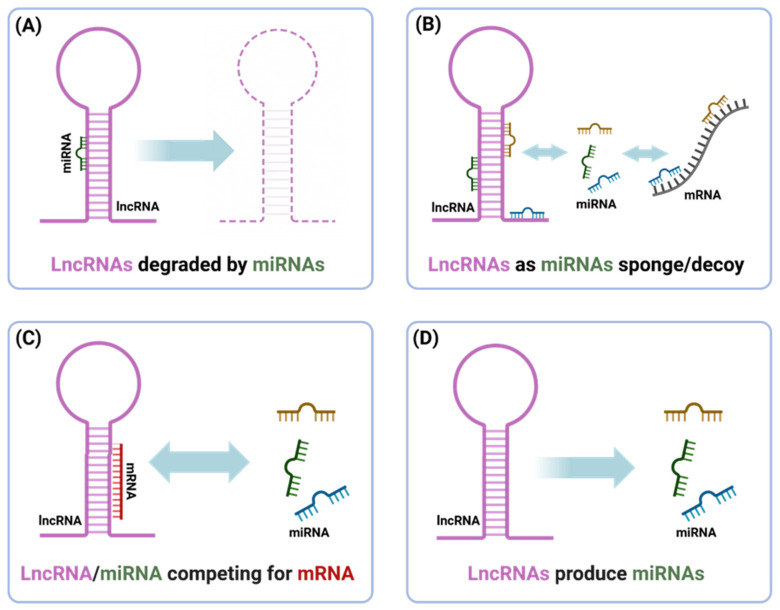
Potential mechanisms of lncRNA–miRNA interactions. (**A**) LncRNAs can be degraded by miRNAs. (**B**) LncRNAs may function as sponges or decoys, sequestering miRNAs and preventing them from binding to their target transcripts. (**C**) LncRNAs and miRNAs can compete for binding sites on shared mRNA targets. (**D**) Some lncRNAs act as precursors and produce functional miRNAs.

Emerging evidence shows that lncRNAs and miRNAs have interdependent regulatory networks that significantly impact cancer immunity. Many lncRNAs perform as competing endogenous RNAs (ceRNAs) that sponge miRNAs and free target transcripts from repression. In cancer immune scenarios, lncRNAs can regulate genes that express factors related to immune evasion, including checkpoint proteins, and immunomodulating cytokines through this mechanism. Growing evidence indicates that lncRNAs and miRNAs interact in several ways to regulate gene expression and cancer immunity. These interactions include lncRNA degradation through miRNAs, lncRNAs functioning as miRNA sponges, lncRNAs competing with miRNAs for mRNA binding, and lncRNAs acting as precursors of miRNAs. In totality, these mechanisms create complicated regulatory crosstalk that extensively impacts tumorigenesis and immune regulation (Figure 10).

In colorectal cancer, for example, lncRNA HOTAIR functions as a sponge for miR-206 and frees CCL2 mRNA from being impacted by miR-206. Following over-expression of CCL2 mRNA, high levels of CCL2 recruit tumor-associated macrophages (TAMs) and reduce anti-tumor immunity. Similarly, lncRNA SNHG17 acts as a sponge for miR-23a-3p and upregulates levels of CXCL12, contributing to an immunosuppressive tumor microenvironment [13]. In general, lncRNAs and miRNAs demonstrate that lncRNA–miRNA signaling modulates immunoregulatory signals and influences immunity toward immune suppression versus immune reactivation, depending on which pathway is being targeted.

A particularly salient axis of this crosstalk is the regulation of immune checkpoints. For example, TUG1 sponges miR-141 and miR-340, resulting in increased PD-L1 and CD47 on tumors, as well as TUG1 binding the transcription factor YBX1, further enhancing the transcription of these immune-evasive signals, thus establishing a self-perpetuating feedback loop that promotes tumor escape [15]. Another interesting example is TINCR, which is activated by IFN-γ/STAT1 signaling in breast cancer. TINCR not only recruits DNMT1 to silence miR-199a-5p but also sponges the remaining cytoplasmic miR-199a-5p, which leads to the upregulation of USP20 and the stabilization of PD-L1. This TINCR–miR-199a–USP20 axis contributes to tumor resistance to T cell killing, whose effects can be reversed by either TINCR knockdown and/or PD-L1 blockade effects [14]. In pancreatic cancer, a similar mechanism is observed, with PMSB8-AS1 sponging miR-382-3p, leading to increases in STAT1 and PD-L1 expression, and again enabling immune escape [142]. Overall, the emergent pattern from this work indicates that lncRNAs often work together with miRNAs to regulate key checkpoint pathways like PD-1/PD-L1 and CTLA-4, which ultimately shape immune surveillance and modulate the response to immunotherapies.

The function of lncRNA–miRNA interactions go far beyond checkpoints; in fact, they play a key role in mediating immune cell polarization and activity within the tumor microenvironment. One prime example of this is with TAMs, in which lncRNAs promote pro-tumoral M2 polarization by sponging miRNAs that suppress the M2 process. In colorectal cancer, the lncRNA, LINC00543, blocks maturation of miR-506-3p, resulting in elevated FOXQ1 and subsequently CCL2, which recruits macrophages and skews macrophages toward the M2 phenotype [143,144,145]. Similarly, MIR155HG acts as a competitive endogenous RNA (ceRNA) for miR-650, to stabilize ANXA2 expression to drive M2 polarization [146]. A third lncRNA, HLA-F-AS1, further promotes the differentiation of M2 macrophages, by sponging the tumor-suppressive miR-375 to upregulate PFN1 levels in macrophages [147]. On the contrary, several lncRNAs serve to promote anti-tumor immunity; NBR2, which is typically downregulated in tumors, serves to sponge miR-19a and release immunosuppressive targets from inhibition; this has the net effect of repolarizing macrophages to an M1 phenotype, and reducing oncogenic HIF-1α and AKT/mTOR signaling to suggest that the restoration of specific lncrRNA–miRNA axes has the potential to bolster host immunity [148].

These ceRNA interactions have also been observed in the regulation of T cell function. LncRNAs can induce T cell apoptosis or dysfunction by sponging the miRNAs that otherwise maintain T cell function. For example, SNHG4 was expressed highly in colorectal cancer, and promotes apoptosis of CD4+ T cells through a miRNA sponge binding to miR-144-3p and competing with the MET oncogene. The outcome was the upregulation of c-Met, involving activation of PD-1/PD-L1 signaling and the exhaustion of helper T cells [149]. Other lncRNAs interfere with cytotoxic T cell functions. For example, ELFN1-AS1 decreased CD8+ T cell cytotoxicity through downregulation of NKG2D and Granzyme B; however, it did this via a miRNA sponge-independent mechanism [150]. Interestingly, H19 in thyroid cancer positively correlated with CD8+ T cells, CD4+ T cells, B cells, and other infiltrates, which indicates that in some contexts, lncRNA-miRNA interactions may increase the recruitment of immune cells [15]. LncRNAs can also modulate T cell differentiation; for example, lnc-SGK1 promotes immunosuppressive Th2/Th17 polarization in gastric cancer, while exosomal CRNDE-h supports Th17 differentiation [151] in colorectal cancer through RORγt. Overall, these studies emphasize that lncRNA–miRNA crosstalk is not limited to a single immune axis, but influences checkpoint regulation, macrophage polarization, T cell apoptosis, and differentiation via more general actions. Depending on the molecular context, these interactions may also inhibit or enhance anti-tumor immunity, making them interesting therapeutic targets for regulating immune responses in cancer.

## 4. Discussion

### 4.1. Future Perspectives

New technologies, such as single-cell RNA sequencing (scRNA-seq) and spatial transcriptomics, are revolutionizing our ability to study thyroid cancer by providing unprecedented opportunities for high-resolution gene expression analysis in the context of individual cells within their associated microenvironments. These cutting-edge techniques show the remarkable heterogeneity of thyroid tumors, including multiple different cell types and their complex partnerships, such as cancer cells, immune cells, and cancer-associated fibroblasts (CAFs). Examining these relationships is essential to our understanding of tumor progression, metastatic spread, and mechanisms of treatment resistance [152,153]. Spatial transcriptomics add yet another layer of complexity, as they couple gene expression with information about their location within the tissue, allowing researchers to understand the influence of the tumor microenvironment on cancer and how this interaction affects treatment response [154]. In personalized medicine, these technologies provide the opportunity to identify unique biomarkers, paired with druggable targets for more effective therapies [155]. One emerging area of interest is the role of lncRNAs as regulators and prospective biomarkers in thyroid cancer [156]. The recent advances in sequencing technologies have led to large-scale sequencing of lncRNAs, which has accelerated their discovery, functional profiling, and potential to act as prognostic markers in thyroid cancer. Both scRNA-seq and spatial transcriptomics will further our understanding of lncRNA function, their involvement in tumor heterogeneity, and their potential as a diagnostic/prognostic tool [153]. Thus, the integration of scRNA-seq and spatial transcriptomics will promote advances in surgical pathology and build momentum for future possibilities associated with precision oncology, which account for the specific molecular and cellular landscape of every patient.

### 4.2. Clinical Evidence and Translational Challenges

While interventional studies of lncRNA-directed therapeutics in thyroid cancer are still in their infancy, new human studies do support a potential clinical role for lncRNAs as biomarkers and possible companion diagnostics. In an observational clinical study of 89 patients with PTC, circulating HOTAIR was significantly higher in those with cervical lymph node metastasis. It was also shown to functionally correlate with a role in inducing increased EMT via Wnt signaling and β-catenin. This suggests that HOTAIR may serve as a serum biomarker for risk stratification and monitoring disease progression in patients with PTC [157].

Although lncRNAs are promising therapeutic targets, their clinical translation has substantial limitations and challenges associated with delivery, specificity, safety, and biological complexity. A major obstacle is the efficient and safe delivery of lncRNA-targeting agents (e.g., antisense oligonucleotides, siRNAs). Delivery systems, such as lipid nanoparticles, viral vectors, and extracellular vesicles, have limitations, including toxicity, immunogenicity, poor stability, and a high cost of production [158,159]. Furthermore, naked lncRNA therapeutics are also vulnerable to rapid degradation in body fluids, thus decreasing their potential in vivo. lncRNAs also often have multiple isoforms and complex secondary structures, making the design of highly specific therapeutics difficult. Off-target effects can lead to unintended and hazardous gene expression and subsequent toxicity, presenting safety concerns for clinical applications [160]. Furthermore, lncRNAs are poorly conserved across species, presenting challenges for preclinical studies and translation from animal models to humans [161]. LncRNA functions are often context-dependent, and many mechanisms remain poorly understood, complicating target selection and validation. Notwithstanding some promising preclinical outcomes, there are very few lncRNA-targeting therapeutics in clinical trials, and none have been approved for any clinical application. Most of the lncRNA-targeting research remains in early human clinical or experimental studies [162].

## 5. Conclusions

In summary, the complex interaction between lncRNAs and the pathogenesis of thyroid cancer highlights the importance of lncRNAs in regulating the processes involved in defined oncogenic processes, which are described by the hallmarks of cancer [21]. LncRNAs play a functional role in sustaining proliferative signaling [41], circumventing growth suppression [22], resistance to apoptosis [47], replicative immortality [53], promoting angiogenesis [25], invasion and metastasis [74], metabolic reprogramming [81], immune evasion [93], inducing genomic instability [29], and tumor-promoting inflammation [101]. The comprehensive account presented in this review emphasizes the various molecular mechanisms by which lncNRAs operate, such as through microRNA binding, modulating critical signaling pathways including PI3K/AKT and the Wnt/β-catenin signaling pathways [41,74], and epigenetic regulation [51]. Altogether, these mechanisms influence tumor progression, metastatic potential, and the development of treatment resistance in thyroid cancer.

Dysregulated lncRNAs will have significant value as clinical biomarkers for a more accurate diagnosis and prognostic assessment of thyroid cancer patients. Their distinct expression profiles associated with clinical parameters and patient outcomes will allow for the personalization of treatment pathways for patients with thyroid cancer. Furthermore, silencing the target lncRNA can be seen as one of the new means of therapeutic intervention following therapy that may overcome the resistance and aggression of the tumor.

At present, there are no approved lncRNA-targeted agents in clinical practice for thyroid cancer or any other malignancy; however, there is rapid advancement in this area with several exciting preclinical and early-clinical strategies being developed. Approaches such as oligonucleotides, antisense oligonucleotides, RNA interference (RNAi), and CRISPR-Cas9 genome editing have been considered to selectively inhibit oncogenic lncRNAs, and reductions in tumor growth, proliferation, and drug resistance have been observed in both in vitro and in vivo models [163]. As an example of this, the lncRNA MALAT1 has been studied as a potential therapeutic target, and in multiple tumor types; MALAT1 inhibition has demonstrated anti-cancer effects, although these strategies are yet to become routine clinical practice [164]. Major challenges hindering clinical translation include targeted delivery, off-target minimization, and ensuring safety and tolerability. Nevertheless, the distinct expression patterns and regulatory specificity of lncRNAs make them attractive and feasible candidates for future molecular therapies, provided that further research and clinical trials confirm their therapeutic potential and safety [163].

Future studies should concentrate on using new technologies such as single-cell RNA sequencing and spatial transcriptomics to further delineate the complex roles of lncRNAs in a more specific way. Incorporating these techniques into a clinical setting will facilitate precision in oncology and further improve our abilities to accurately diagnose, prognosticate, and treat thyroid cancer.

## Figures and Tables

**Table 1:** LncRNAs in papillary thyroid carcinoma (PTC) and their hallmark-related roles.

LncRNA	Hallmark Affected	Mechanism	Experimental Model	Main Findings	Ref.
MEG3	Invasion and Metastasis; TME/CAF remodeling	Tumor-suppressive; stromal-enriched MEG3 in CAFs associated with LNM; post-transcriptional repression of Rac1 (3′UTR)	Bulk and single-cell RNA-seq; in vitro	MEG3 signal predicts lymph node metastasis and CAF infiltration; overexpression suppresses migration/invasion via Rac1 downregulation	[31,32]
H19	Proliferation, Apoptosis, Angiogenesis, Immune Infiltration	Activates PI3K/AKT; ↑ VEGF; ↑ immune infiltration	In vitro; tissues	Overexpression linked to increased proliferation/apoptosis resistance and immune cell infiltration signatures in thyroid carcinoma	[41,61]
KCNQ1OT1	Immune Evasion	Sponges miR-15a → ↑ PD-L1	In vitro	Oncogenic; promotes growth and motility via miRNA sponging to de-repress target oncogenes	[93]
GAS5	Apoptosis, Therapy Sensitivity	Sponges miR-362-5p → ↑ SMG1; Akt/mTOR inhibition	In vitro	Tumor-suppressive; enhances apoptosis and radioiodine sensitivity	[50]
SOCS2-AS1	Proliferation, FAO	Degrades p53; ↑ fatty acid oxidation	In vitro	Oncogenic; promotes proliferation and metabolism	[43]
ZFAS1	Proliferation, EMT	Repressed by p53; regulates miRNAs	In vitro	Oncogenic; promotes proliferation/invasion	[22]
HOTTIP	Apoptosis Resistance	Sponges miR-744-5p → ↑ c-Myc	In vitro	Oncogenic; inhibits apoptosis	[46]
RUNDC3A-AS1	Proliferation, Apoptosis	miR-151b/SNRPB axis	In vitro	Oncogenic; promotes growth, inhibits apoptosis	[47]
RPL34-AS1	Apoptosis	Modulates miRNAs	In vitro	Tumor-suppressive; induces apoptosis	[48]
ATP1A1-AS1	Apoptosis	Regulates downstream effectors	In vitro	Tumor-suppressive; apoptosis induction	[49]
TUG1	Replicative Immortality	↑ TERT	In vitro	Oncogenic; promotes telomerase activity	[56]
FOXD2-AS1	Replicative Immortality	Sponges miR-7-5p → ↑ TERT	In vitro	Oncogenic; enhances immortality	[56]
ACVR2B-AS1	Replicative Immortality	Sponges miR-195-5p → ↑ FGF2	In vitro	Oncogenic; proliferation, poor prognosis	[58]
ABHD11-AS1	Proliferation, Angiogenesis	Modulates PI3K/Akt, EMT	In vitro; tissues	Oncogenic; angiogenesis and proliferation	[61]
PTCSC3	Invasion, Angiogenesis	Suppresses Wnt/β-catenin	In vitro	Tumor-suppressive; inhibits EMT and angiogenesis	[63]
ANRIL	Angiogenesis	Activates NF-κB → ↑ VEGF	Rat model; cross-cancer	Oncogenic; angiogenesis	[66]
AFAP1-AS1	Angiogenesis, Stemness	Sponges miR-27b-3p → ↑ VEGF-C	In vitro	Oncogenic; CSC properties, angiogenesis	[67]
SNHG1	Angiogenesis	HIF-1α/VEGF axis	In vitro (hypoxia)	Oncogenic; enhances angiogenesis	[68]
BANCR	EMT, Invasion	MAPK regulation	In vitro	Oncogenic; promotes invasion	[70]
DOCK9-AS2	EMT, Stemness	SP1 binding; sponges miR-1972 → ↑ CTNNB1	In vitro; exosomes	Oncogenic; EMT, stemness, metastasis	[74]
PVT1	Metabolism, Angiogenesis	Stabilizes STAT3; glycolysis regulation	In vitro	Oncogenic; glycolysis and VEGFA activation	[27,65]
LINC00671	Metabolic Reprogramming	Downregulates LDHA	In vitro	Tumor-suppressive; suppresses glycolysis	[84]
GLTC	Metabolic Reprogramming	LDH1 succinylation → ↑ lactate	In vitro	Oncogenic; drives glycolysis, radioiodine resistance	[85]
HOTAIR	Immune Evasion	↑ PD-L1; exosomal	In vitro	Oncogenic; T cell suppression	[92]
NKILA	Immune Evasion	Induces CTL/T helper AICD	In vitro	Oncogenic; immune escape	[91]
MIAT	Immune Evasion, Prognosis	↑ PD-1/PD-L1/CTLA4; EZH2 axis	In vitro; tissues	Oncogenic; poor survival and immune suppression	[19,94]
SLC26A4-AS1	Genome Instability	Alters MRN complex → instability	In vitro	Tumor-suppressive; loss → metastasis	[29]
CamK-A	Inflammation, Angiogenesis	Activates PNCK → NF-κB	In vitro	Oncogenic; promotes inflammatory TME	[103,104]
LINC00887	Proliferation, Invasion; Immune Evasion	Activates Wnt/β-catenin and Hippo; upregulates PD-L1	In vitro (CRISPR knockdown)	Oncogenic; promotes PTC growth, invasion, and immune evasion via Wnt/Hippo and PD-L1 upregulation.	[115]
LIFR-AS1	Proliferation, Angiogenesis, Invasion	Sponges miR-31-5p to upregulate SIDT2	In vitro; in vivo (xenograft)	Tumor-suppressive; inhibits proliferation and angiogenesis by sponging miR-31-5p and upregulating SIDT2.	[116]
ASMTL-AS1	Proliferation; Metabolic Reprogramming	Sponges miR-93-3p and miR-660 to upregulate FOXO1	In vitro	Tumor-suppressive; inhibits growth and glycolysis via miR-93-3p/miR-660/FOXO1 axis.	[117]
RP11-547D24.1	Proliferation, Apoptosis, Invasion	Unclear; tumor suppressor via TCGA analysis	In vitro (loss-of-function)	Tumor-suppressive; loss enhances proliferation and invasion while reducing apoptosis.	[118]
lnc-MPEG1-1	Proliferation, Metastasis	Sponges miR-766-5p	In vitro; clinical samples	Oncogenic; promotes proliferation and metastasis by sponging miR-766-5p.	[119]
GHET1	Proliferation, Invasion	Modulates cell-cycle/apoptosis; exact target unclear	In vitro; clinical samples	Oncogenic; enhances invasion and proliferation, linked to aggressive features.	[120]
UCA1	Proliferation	Sponges miR-204 to upregulate BRD4	In vitro; in vivo (mice)	Oncogenic; promotes cell proliferation via miR-204/BRD4 axis.	[121]
DUXAP8	Proliferation, Invasion	Sponges miR-223-3p to upregulate CXCR4	In vitro	Oncogenic; sponges miR-223-3p to increase CXCR4, promoting invasion and proliferation.	[122]
PAPAS	Proliferation	Downregulates oncogenic HOTTIP	In vitro; patient tissues	Tumor-suppressive; represses HOTTIP to inhibit proliferation.	[123]
LINC00924	Proliferation, Invasion; Apoptosis	ICD-related; may impact immune microenvironment	In vitro; bioinformatic analysis	Tumor-suppressive; reduces proliferation, invasion, and increases apoptosis; may affect immune microenvironment.	[124]
SNHG3	Proliferation, Metastasis	Inhibits AKT/mTOR/ERK pathway	In vitro; in vivo	Tumor-suppressive; inhibits growth and metastasis by regulating AKT/mTOR/ERK.	[125]
AK023507	Proliferation, Metastasis	Inhibits Wnt/β-catenin signaling	In vitro	Tumor-suppressive; downregulates Wnt/β-catenin to inhibit proliferation and invasion.	[126]
LINC01614	Proliferation, Invasion	Genomic instability-related; modulates TME	In vitro; patient data	Oncogenic; promotes PTC progression and alters tumor microenvironment.	[18]
LINC00704	Proliferation, Invasion	Sponges miR-204-5p to upregulate HMGB1	In vitro	Oncogenic; drives growth and motility via miR-204-5p/HMGB1 axis.	[127]
lncRNA BRM	Proliferation, Invasion	Sponges miR-331-3p to upregulate SLC25A1	In vitro	Oncogenic; promotes PTC progression via miR-331-3p/SLC25A1 axis.	[128]
CCHE1	Proliferation, Invasion	Activates ERK/MAPK pathway	In vitro; patient tissues	Oncogenic; activates ERK/MAPK pathway, promoting aggressive features.	[129]

↑ indicates upregulation or activation.

**Table 2:** Clinical implications of dysregulated lncRNAs in thyroid cancer, including their potential as diagnostic or prognostic biomarkers and therapeutic targets.

LncRNA	Clinical Application	Expression Pattern	Patient Correlation	Diagnostic/Prognostic Value	Therapeutic Targeting	Ref.
ACVR2B-AS1	Prognostic biomarker	Upregulated	High expression correlates with metastasis and poor survival	Elevated levels predict poor prognosis	siRNA knockdown affects miR-195-5p and FGF2 pathway	[58]
LUCAT1	Prognostic biomarker and therapeutic target	Upregulated	Associated with advanced tumor stage and poor survival	Independent predictor of poor outcome	siRNA knockdown inhibits JAK-STAT pathway and tumor proliferation	[137]
SLC26A4-AS1	Prognostic marker for metastasis	Downregulated	Low expression linked to lymph node metastasis and poor survival	Low levels predict higher metastatic risk and poor prognosis	Restoration may suppress EMT via DDX5 degradation	[29]
LINC00284	Tumor suppressor; prognostic marker in panel	Downregulated	High expression associated with improved survival	Included in 3-lncRNA prognostic signature	Potential modulation via miR-205/E2F1 axis	[138]
LINC00704	Prognostic biomarker and therapy target	Upregulated	High expression associated with aggressiveness and shorter survival	Independent predictor of poor outcome	siRNA-mediated silencing reduces cell growth and induces apoptosis	[97]
H19	Diagnostic biomarker and immune infiltration marker	Downregulated	Loss of expression linked to poor OS; high expression linked to increased immune infiltration	Diagnostic and prognostic implications based on expression and immune infiltration	Suggested restoration or demethylation may be beneficial	[139]
lnc-MPEG1-1	Diagnostic and predictive lncRNA	Upregulated	High expression correlates with lymph node metastasis	Part of a predictive nomogram for lymph node metastasis	Knockdown inhibits EMT via miR-766-5p axis	[119]
XIST	Therapeutic and diagnostic target	Upregulated	High expression positively correlates with MET and tumor progression	High XIST expression marks aggressive tumors	Silencing restores miR-34a and suppresses MET/PI3K/AKT pathway	[140]
LINC02471 and DOCK9-DT	Prognostic and immune-related markers	Upregulated	High expression associated with immune cell infiltration and risk scoring	Included in a prognostic model (m6A-related lncRNAs)	No direct targeting; potential through m6A modulation	[141]
MIAT	Prognostic biomarker and therapeutic target	Upregulated	High expression correlates with EZH2 and poorer survival	Predicts recurrence and overall survival	Knockdown affects miR-150-5p/EZH2 axis	[19]

## Data Availability

No new data were created or analyzed in this study. Data sharing is not applicable to this article.

## Abbreviations

The following abbreviations are used in this manuscript:

ATC	Anaplastic Thyroid Carcinoma
ASO	Antisense Oligonucleotide
ceRNA	Competing Endogenous RNA
CRISPR	Clustered Regularly Interspaced Short Palindromic Repeats
CTLA-4	Cytotoxic T-Lymphocyte–Associated Protein 4
EMT	Epithelial–Mesenchymal Transition
ERK	Extracellular Signal-Regulated Kinase
FTC	Follicular Thyroid Carcinoma
HIF	Hypoxia-Inducible Factor
IL-6	Interleukin-6
lncRNA	Long Non-Coding RNA
MAPK	Mitogen-Activated Protein Kinase
miRNA	microRNA
MTC	Medullary Thyroid Carcinoma
NF-κB	Nuclear Factor Kappa-Light-Chain-Enhancer of Activated B Cells
PD-1	Programmed Cell Death Protein 1
PD-L1	Programmed Death-Ligand 1
PI3K	Phosphoinositide 3-Kinase
PTC	Papillary Thyroid Carcinoma
scRNA-seq	Single-Cell RNA Sequencing
STAT3	Signal Transducer and Activator of Transcription 3
TAM	Tumor-Associated Macrophage
TC	Thyroid Cancer
TERT	Telomerase Reverse Transcriptase
TME	Tumor Microenvironment
VEGF	Vascular Endothelial Growth Factor
Wnt	Wingless/Integrated
